# High Sensitivity Determination of TNF-α for Early Diagnosis of Neonatal Infections with a Novel and Reusable Electrochemical Sensor

**DOI:** 10.3390/s17050992

**Published:** 2017-05-10

**Authors:** Liangliang Li, Miaomiao Li, Wenwen Wang, Qian Zhang, Dongyun Liu, Xianghong Li, Hong Jiang

**Affiliations:** 1 Department of Neonatology, The Affiliated Hospital of Qingdao University, Qingdao 266000, China; cmuliliang@163.com (L.L.); liudongyun007@163.com (D.L.); lixianghong0329@126.com (X.L.); 2 Center for Medical Research, The Affiliated Hospital of Qingdao University, Qingdao 266000, China; lmm170810553@163.com (M.L.); zhangqian0535@sina.com (Q.Z.); 3 Intensive Care Unit, The Affiliated Hospital of Qingdao University, Qingdao 266000, China; wangwenwen23@126.com

**Keywords:** neonatal infections, TNF-α, aptamers, reuse, chain replacement reaction

## Abstract

Early diagnosis is vital for the reduction of mortality caused by neonatal infections. Since TNF-α can be used as a marker for the early diagnosis, the detection of TNF-α with high sensitivity and specificity has great clinical significance. Herein, a highly sensitive and reusable electrochemical sensor was fabricated. Due to the high specificity of aptamers, TNF-α could be accurately detected from five similar cytokines, even from serum samples. In addition, Au nanoparticles (AuNPs) with a high surface area were able to combine a large number of doxorubicin hydrochloride (DOXh), which made the sensor have a high sensitivity. The sensor had a good linear relationship with TNF-α concentration in the range from 1 to 1 × 10^4^ pg/mL and the lowest detection limit is 0.7 pg/mL. More important was that the sensor could be reused 6 times by a crafty use of chain replacement reaction. Meanwhile, the detection time and cost were greatly reduced. Thus, we believe that these advantages of higher specificity and sensitivity, lower cost, and shorter detection time will provide a stronger potential for early diagnosis of neonatal infections in clinical applications.

## 1. Introduction

Neonatal infections are the most common causes of neonatal morbidity worldwide [1,2,3]. It is estimated that 400,000 newborns die from serious infections annually [4]. With the indistinguishable clinical symptoms and the rapid development, neonatal infections are prone to misdiagnosis and thus delayed treatment [5]. Thus, the technology for early diagnosis of neonatal infections is of great significance. Considering that the pro-inflammatory cytokine TNF-α plays an important role in the inflammation induced by infections, and the concentration of TNF-α in serum is associated with the degree of infections, TNF-α can be a good indicator for the early diagnosis of neonatal infections [6,7,8]. Since TNF-α levels (60–100 pg/mL) are very low in the early stage of neonatal infections, the sensitivity and specificity of the detection methods are strictly required.

Until now, the commonly used detection methods for TNF-α that have been reported are ELISA [9], radioimmunoassays [10], and time-resolved fluorescence assays [11]. However, most of these methods are costly and time-consuming, have low sensitivity and specificity, or need professional operators. These shortcomings limit their use in TNF-α detection of newborns. Compared with these traditional methods, electrochemical biosensors are more competent for the detection of TNF-α because of its distinct advantages [12,13,14,15]. Firstly, the molecular specific recognition mechanism improves selectivity and specificity. Secondly, a high flux, a fast response, and little sample consumption enhance efficiency and sensitivity. Thirdly, miniaturized equipment and low costs ensure that it is easily operated and generalized. In view of these advantages, many studies have focused on the application of electrochemical biosensors in the detection of TNF-α, and some have been reported. Mi Kyoung Park et al. fabricated an EIS-based biosensor for the ultra-sensitive detection of TNF-α from non-diluted human serum [16]. Luis Bujanda et al. used a proximity ligation assay for the quantification of TNF-α [17]. Mohammad Mazloum-Ardakani et al. synthesized Ag@Pt core-shell nanoparticles for the detection of TNF-α [18]. Although all these methods have achieved good results, there is still space for further improvement in some aspects, including sensitivity, cost, and time consumption. 

**Scheme 1:**
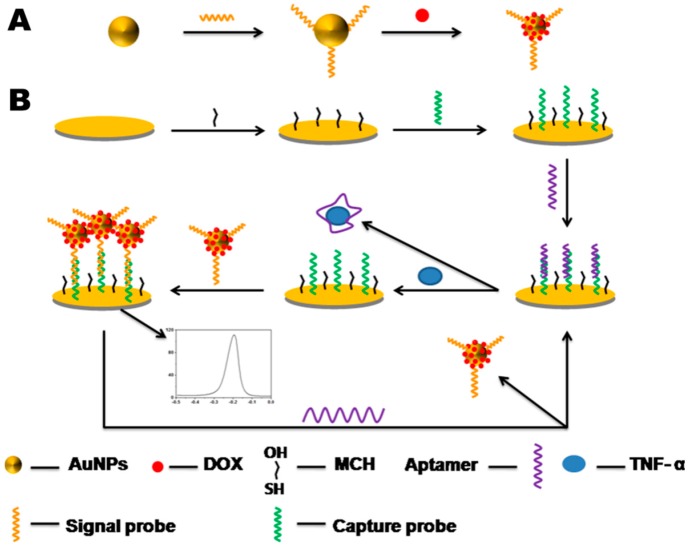
(**A**) Synthesis of AuNPs@DOX signal probes (**B**). The fabricating procedure and reuse of TNF-α electrochemical sensor and the detection of TNF-α.

As a good bio-composite nano-material, Au nanoparticles (AuNPs) can greatly increase the current response of the modified sensor with a good conductive ability. Moreover, with a substantial specific surface area, AuNPs can also largely immobilize the DNA probes via Au-S bonds and signal molecule [19,20]. Doxorubicin hydrochloride (DOXh) is not only a kind of anthracycline anti-tumor drug but is also strong and stable electro-active [21]. According to this, DOXh was selected to be a signal molecule. A large number of positively charged DOXh could be bound to the surface of negatively charged AuNPs by an electrostatic adsorption effect for the amplifying signal (Scheme 1A).

It is widely known that polishing gold electrodes and immobilizing DNA probe is a time-consuming progress in the traditional electrochemical sensor. In order to solve this shortcoming, some studies have designed a number of methods for the reuse of electrodes. For example, Prof Genxi Li’s team used magnetic beads to fabricate a magneto-controlled moveable architecture in February 2014. It was found that the sensor could be reused three times [22]. Prof Wenli Feng’s team utilized a Uracil Excision Enzyme to realize eightfold reuse of a cyto-sensor in March 2016 [23]. These two methods realized the reuse of a sensor, but the detection cost could be further decreased. For that, the nucleic acid strand displacement reaction was used (Scheme 1B). The method not only achieved the reuse of the sensor, but also further decreased the loss of capture probes and aptamers. 

Generally, many traditional electrochemical sensors use antibodies to capture TNF-α or other antigen and achieved very good results. However, the cost of the antibody is very high. To reduce costs, some researchers have proposed aptamers and ligands. An aptamer is a kind of oligonucleotide sequence, which is screened by SELEX (Systematic Evolution of Ligands by Exponential Enrichment). It can bind to target molecule with high affinity and specificity [24]. More importantly, the cost of aptamers is very low. 

In our study (Scheme 1B), after 6-mercapto-1-hexanol (MCH) was immobilized on the gold electrode, the capture probes were inserted in the gap between two MCH molecules. The method could make the DNA distribution more uniform and the signal more stable [25]. AuNPs and DOXh were used for improving the sensitivity. Moreover, the aptamer was chosen for binding TNF-α with high affinity, high specificity, and low cost. Most importantly, we realized the reuse of the sensor by DNA displacement reaction. Compared with the reported TNF-a sensor, our sensor has greater sensitivity and specificity, a lower cost, and a shorter detection time. We believe that this TNF-α sensor will have superior performance in real blood samples and has a strong potential for clinical applications.

## 2. Materials and Methods 

### 2.1. Reagents and Materials

TNF-α was purchased by Solarbio Inc. (Shanghai, China). 6-mercapto-1-hexanol (MCH) come from Sigma Chemical Co, St. Louis, MO, USA. All DNA chains were synthesized by Sangon Inc. (Shanghai, China), and their sequences are shown in Table S1. All oligonucleotides stock solutions (10 μM) were prepared with TE buffer solution (10 mM Tris-HCl, 1.0 mM EDTA, pH 7.5) and kept frozen at −20 °C. Tris-HCl with a concentration of 10 mM (pH 7.4), containing 10 mM KCl and 10 mM MgCl_2_, was the binding buffer. Tris-HCl buffer with a concentration of 20 mM (pH 7.4) containing 0.1 M NaCl, 5.0 mM MgCl_2_, and 0.005% Tween−20 as well as a 0.01 M phosphate buffer solution (PBS, pH 7.4) were used as washing buffer. The working solution of DPV was 10 × PBS. The working solution of CV and EIS contained 5 mM [Fe(CN)6]^3−/4−^ and 0.1 M KCl. Water (resistivity, 18.2 MΩ) was purified using the Millipore-Q water purification system.

### 2.2. Instrumentation

A CHI660D electrochemical workstation (Shanghai Chenhua Instruments, Shanghai, China), a UV transilluminator (Bio-Rad Laboratories, Hercules, CA, USA), a Zetasizer Nano ZS90 (Malvern Instruments Ltd., Malvern, UK), and transmission electron microscopy (TEM, H600, Hitachi, Japan) were used to investigate the morphology of AuNPs. All electrochemical measurements were carried out by a conventional three-component electrochemical system, including a modified gold electrode as the working electrode, a KCl-saturated Ag/AgCl electrode as a reference electrode, and a Pt wire as the auxiliary electrode. In addition to the special requirements, most experiments were performed at room temperature (25 ± 2 °C).

### 2.3. Preparation of Gold Electrode

The gold electrode (3 mm diameter) was carefully polished to a mirror-like finish by 0.3 and 0.05 μm alumina powder, and then they went through successive sonication for 10 min in ultrapure water, ethanol, and ultrapure water, respectively. Finally, it was activated in freshly prepared piranha solution (98% H_2_SO_4_:30% H_2_O_2_, 3:1 by volume) for 5 min and rinsed with ultrapure water thoroughly [26].

### 2.4. Preparation of the AuNPs

Sixteen nanometers of AuNPs were prepared according to a reported method. Briefly, after 50 mL of HAuCl4 solution (0.01%, *m/v*) was boiled via vigorous stirring, 2 mL of trisodium citrate solution (1%, *m/v*) was added quickly into the boiling solution. When the color of the solution changed from pale yellow to wine red, the AuNPs were formed. The resulting solution was cooled to room temperature and stored in brown glass bottles at 4 °C for future use [27,28,29].

### 2.5. Preparation of the Signal Molecule

Firstly, according to the previously reported methods with little modification, a signal probe (T3) modified AuNPs [30]. Before T3 loading on the AuNPs surface, the disulfide bond of thiolated modified T3 was reduced by a 100-fold excess TCEP (tris(2-carboxyethyl) phosphine) at room temperature for 1 h. Then, 10 μL of 100 μM T3 was mixed with 1 mL of an AuNP solution and stirring at 4 °C for 12 h. After that, 10 mL of 1% SDS was added to stabilize the AuNPs with shaking at room temperature for 1 h. Then, 50 mL of a 0.5 M NaCl solution was slowly added for aging the AuNPs-T3. The AuNPs-T3 solution was centrifuged at 12,000 rpm for 5 min and washed 3 times by a 0.01 M phosphate buffer solution (PBS) (pH 7.4). Secondly, the prepared AuNPs-T3 was incubated with 0.05 mM DOX and stirring in the dark for 12 h. Next, the AuNPs-T3@DOX solution was also centrifuged at 12,000 rpm for 5 min and washed 3 times by 0.01 M PBS. Finally, the AuNPs-T3@DOX solution was stored at 4 °C for standby applications.

### 2.6. Fabrication of the TNF-α Sensor

When the gold electrode was cleaned and prepared, 10 μL of 10 μM MCH which is prepared in 75% ethanol was dropped onto the gold electrode and incubated 20 min at room temperature. After rinsed with ultrapure water, 10 μL of 500 nM thiolated capture probe (disulfide bonds reduced by TCEP for 120 min at room temperature in the dark) was inserted and incubated in MCH interspaces for 12 h at 4 °C. After extensively washing with 20 mM Tris-HCl buffer, 10 μL of 100 nM aptamer was subsequently dropped and hybridized with the capture probe for 2 h at 37 °C. After being rinsed with 20 mM Tris-HCl buffer to remove the unhybridized aptamer, TNF-α was added, and the aptamer was then captured from the capture probe by immersing the electrode into a 0.01 M PBS buffer containing different concentrations of TNF-α at 37 °C for 2 h.. Subsequently, the electrode was thoroughly rinsed with 0.01 M PBS. Finally, the signal molecule was dropped, and the electrochemical signal in the work solution was detected.

### 2.7. Electrochemical Detection and Recycling of TNF-α Sensor

The DPV signals of DOX were detected in 10 × PBS. The signals of CV and EIS were detected in the working solution (0.1 M PBS, pH 7.4) containing 5 mM [Fe(CN)_6_]^3−/4−^ and 0.1 M KCl by EIS within the frequency range of 10^−1^ to 1 × 10^5^ Hz with a signal amplitude of 5 mV [31,32,33]. The TNF-α sensor was regenerated by adding 1 μL of aptamer and incubating for 2 h at 37 °C. After that, the electrode was rinsed with ultrapure water (about 20 s) and dried under a stream of nitrogen gas. Finally, the regenerated sensor was ready for the next cycle. 

### 2.8. The Preparation of the Serum

In the first, a certain proportion of anticoagulant was added in blood. Anticoagulant and blood in a 1:9 ratio was added to a certain amount of blood after mixing, centrifugal (3000 rpm, 5–10 min). After that, the supernatant was plasma.

### 2.9. Experimental Protocol

Firstly, MCH was fixed on the gold electrodes for capture probes inserting in the slides of MCH, so that capture probes could stand on the electrodes and dispute more firmly. Secondly, aptamer was adding and hyberized with capture probe. This method could close free capture probes and avoid signal probes non-specifically binding with capture probes. As for the aptamer, TNF-α could specifically bind with aptamers and displace it from the capture probe. Thus, some free capture probes were exposed. Finally, signal probes were incubated with free capture probes and detected by DPV. Moreover, after detection, aptamer was added for displacing signal probes, due to the affinity between aptamer and capture probes stronger than between capture probes and signal probes. This method could help the sensor recover to an original state for the reuse of the sensor.

## 3. Results and Discussion

### 3.1. Characterization of AuNPs and Signal Molecule

**Figure 1:**
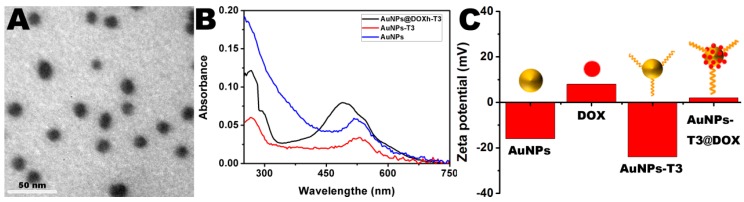
(**A**) TEM images of AuNPs. (**B**) UV–vis absorption spectra of differently surface-functionalized AuNPs. (**C**) Zeta potential analysis of differently surface-functionalized AuNPs.

A transmission electron microscope (TEM) was used to characterize the morphology of AuNPs. As shown in Figure 1, the size of AuNPs was about 16–20 nm. Moreover, AuNPs were distributed evenly without aggregation. Uniform distribution lays the foundation for a stable signal. 

To prove that T3 and DOXh can be stably combined with AuNPs, UV-Vis absorption assays were adopted (Figure 1B). The absorption peak of the AuNPs was about 520 nm. However, when T3 was combined with AuNPs, the absorption had a red shift from 520 to 529 nm, which might be owed to the interparticle plasmon coupling. The red shift and the new absorption peak at 260 nm proved that DNA has successfully combined with AuNPs. Compared with the red line, the black line increased two absorption peaks of DOXh at 290 nm and 480 nm. Thus, the results indicated that AuNPs-T3@DOXh nano-composite had been successfully synthesized

Zeta potential analysis was used to further prove the successful combination of the nano-composite. Figure 1C shows that the surface potentials of AuNPs and DOX were −16 mV and 8 mV, respectively. After AuNPs were combined with T3, the surface potential decreased to −26 mV. This is because that DNA chains were negatively charged. When combined with positively charged DOX, the surface potential increased to 3 mV. These results also demonstrated the successful assembly of AuNPs-T3@DOX.

**Figure 2:**
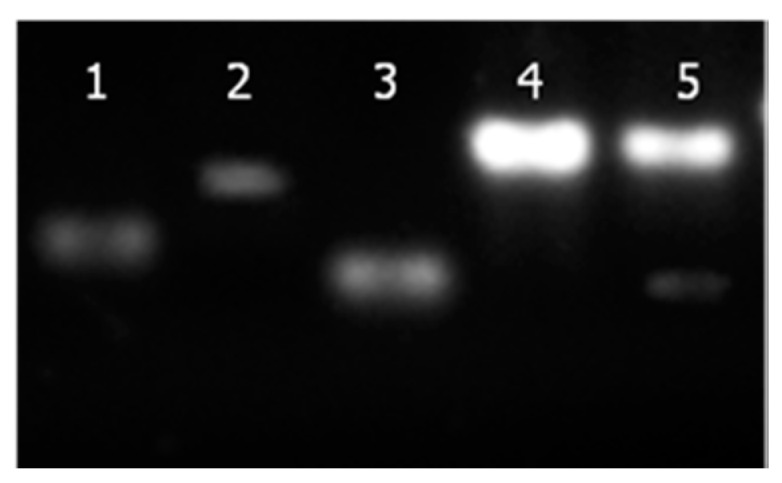
Gel electrophoresis characterization of the prepared cytosensor: Capture probe (T1, Lane 1); aptamer (T2, Lane 2); signal probe (T3, Lane 3); capture probe (T1) plus aptamer (T2) incubated for 2 h at 37 °C (Lane 4); after Capture probe (T1) hybridized with signal probe (T3), aptamer (T2) was added (Lane 5).

To further prove the feasibility of the method, AGE (agarose gel experiment) was used. As shown in Figure 2, the first three lanes are three DNA single chains (T1, T2, and T3). After T1 incubated with T2 for 120 min, there was a bright band at Lane 4. It proved that T1 can hybridize with T2. Lane 5 indicated that aptamer (T2) can displace a capture probe (T1) from a signal probe (T3). The result laid a solid foundation for the reuse of the sensor.

**Figure 3:**
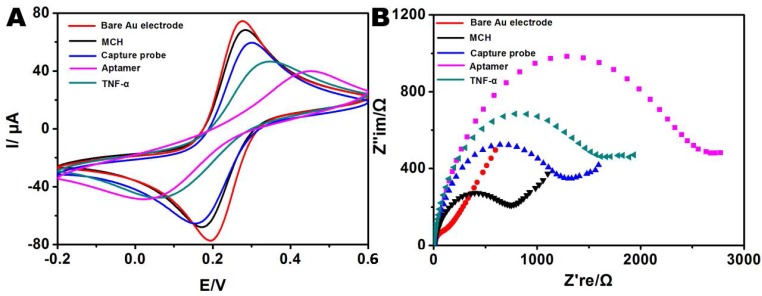
Electrochemical characterization of the TNF-α sensor: (**A**) Cyclic voltammetry and (**B**) Nyquist plots obtained from different electrodes.

Cyclic voltammetry (CV) and electrochemical impedance spectroscopy (EIS) are effective in demonstrating each step of modification of the working electrode. CV detected the current of the gold electrode in the working solution at a scan rate of 0.1 V·s^−1^. Moreover, EIS was used to measure charge-transfer resistance. As shown in Figure 3A, the current decreased when MCH was fixed on the bare electrode, which was attributed to the blocking effect of the MCH. Since the capture probe and aptamer could hinder the electron transfer and the electrostatic repulsion between the negatively charged deoxyribose-phosphate backbones of the capture probe and [Fe(CN)_6_]^3−/4−^, the current suffered an obvious decrease. However, the current increased when TNF-α displaced the aptamer from the capture probe. Moreover, as the other good method of detecting changes in electrode surfaces, EIS was also used to prove the progress. As shown in Figure 3B, after MCH, the capture probe, and the aptamer were fixed on the bare electrode, the value of EIS increased. Thus, the results corroborated the CV results. In sum, the self-assembly of the electrode was successful.

### 3.2. Optimization of Experimental Parameters

**Figure 4:**
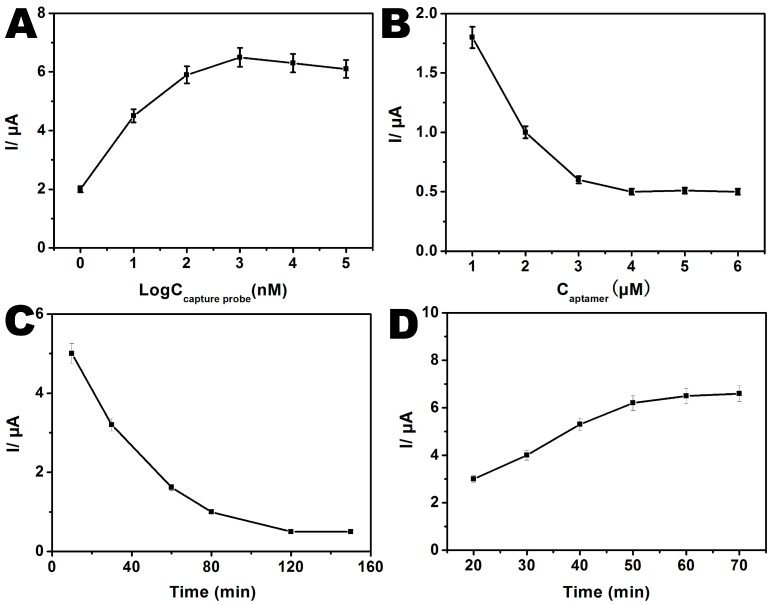
Optimization of four important parameters: concentration of (**A**) capture probe and (**B**) aptamer; (**C**) the hybridization time of the capture probe and aptamer; and (**D**) the incubation time of TNF-α. All data are shown as mean ± S.D.; *n* = 5 per group.

Before detection, we optimized five important parameters for obtaining a better performance. They are the concentrations of capture probe and aptamer, the hybridization time of the capture probe and aptamer, and the incubation time of TNF-α, respectively. Figure 4A showed that DPV signal increased with the concentration of capture probe. However, the DPV signal decreased when the concentration was higher than 1 μM. The account for this phenomenon is that the number of probes is too high to cause spatial rejection of the target DNA. The hybridization efficiency is thus affected. Thus, the best concentration of the capture probe was 1 μM. To decrease the background signal, the effect of the aptamer concentration was studied in the range from 1 to 6 μM (Figure 4B). The background signal gradually decreased from 1 to 3 μM and reached plateaus at 3 μM. Hence, a concentration of 3 μM was chosen in this study. In order to make the hybridization of the capture and probe more thorough, the incubation time was optimized. As shown in Figure 4C, the background signal was lowest after the aptamer and capture probe incubated for 2 h. Moreover, more aptamers was replaced by TNF-α, more signal probe was captured, and DPV signal was higher. Figure 4D showed that the signal was highest after aptamer incubation with TNF-α for 1 h.

### 3.3. Detection Sensitivity and Selectivity

**Figure 5:**
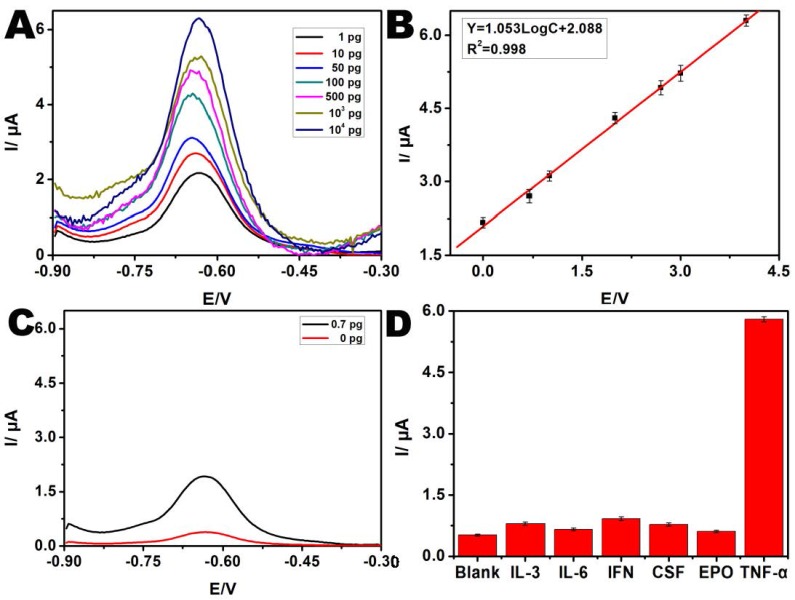
(**A**) DPV responses of the sensor incubated with TNF-α of different concentrations in 1.0 × 10^−2^ M phosphate buffer (pH 7.5) (from 1 pg to 1 × 10^4^ pg/mL). (**B**) Calibration curve of the sensor for TNF-α. (**C**) DPV responses of the lowest concentration of TNF-α (0 and 0.7 pg/mL). (**D**) Specificity of the proposed sensor. All data are shown as mean ± S.D.; *n* = 5 per group.

After Optimization of experimental parameters, different concentrations TNF-α standard samples were detected by this sensor. From Figure 5A, we could found that the signal increased with the concentration of TNF-α. Moreover, there was a good linear relationship between the DPV signal and the logarithm values of the number of TNF-α in the range from 1 pg/mL to 1 × 10^4^ pg/mL. The linear regression equation was Y = 1.053LogC + 2.088, with a correlation coefficient R^2^ = 0.998 (Figure 5B), and the detection limit was 0.7 pg/mL (LOD = 3.3 × SD/Slope) [34]. Moreover, the detection limit concentration was also detected by the sensor (Figure 5C). The results proved that the sensor indeed could enable the detection of the lowest concentration. 

In order to verify the specificity of the method, seven groups of control experiments were set up (Figure 5D). The sensor’s responses to six different cell factors, respectively, including IL-3, IL-6, IFN, CSF, EPO, and TNF-α, were observed. Stronger signals are generated only when TNF-α exists. However, like the blank, other cell factors could not generate stronger signals. These DPV responses indicated that the proposed electrochemical biosensor displayed high specificity and good sensitivity for the detection of TNF-α.

A comparison of recently reported biosensors was shown in Table S2. The comparison indicated that our sensor is a very good biosensor for the detection of TNF-α.

### 3.4. Reproducibility and Stability of the Sensor

**Figure 6:**
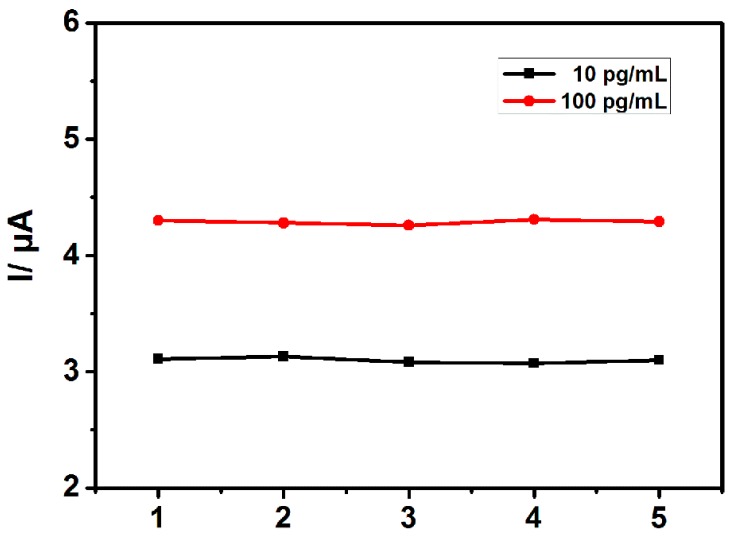
The electrode-to-electrode reproducibility.

As a good sensor, reproducibility and stability are also important, except good sensitivity and specificity. Thus, the electrode-to-electrode reproducibility was tested. Five sensors were prepared with the same treatment for testing 10 and 100 pg/mL of TNF-α, and the relative standard deviation (RSD) of 1.38% and 1.92% were obtained (Figure 6). The data suggested that the method has excellent reproducibility.

The stability of the sensor was also investigated. After the cytosensor was stored in a refrigerator at 4 °C for 20 d, 90.8% of the DPV signal was retained. Therefore, our sensor has a satisfactory stability.

### 3.5. Reusing of the Sensor

**Figure 7:**
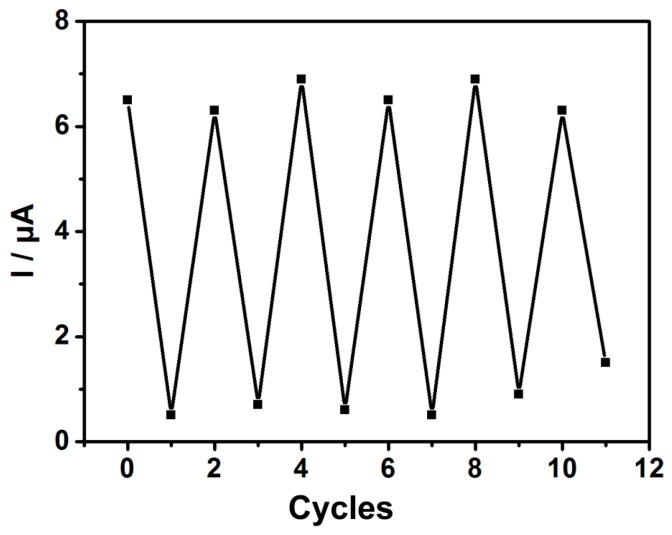
The sensor was circled six times.

Since traditional sensors cannot be recycled, they are costly and time-consuming. In order to solve this problem, we skillfully used the chain substitution reaction. After detection, adequate aptamer was used to displace the signal molecule from the capture probe on the electrode. There were then only capture probes on the electrode, and the initial state was restored. Hence, the next detection could be restarted. For proving the sensor could be reused, a recycle experiment was designed. After six cycles, the background signal increased (Figure 7). The result indicated that the sensor could be reused up to six times.

### 3.6. Detection of the Serum Samples

**Figure 8:**
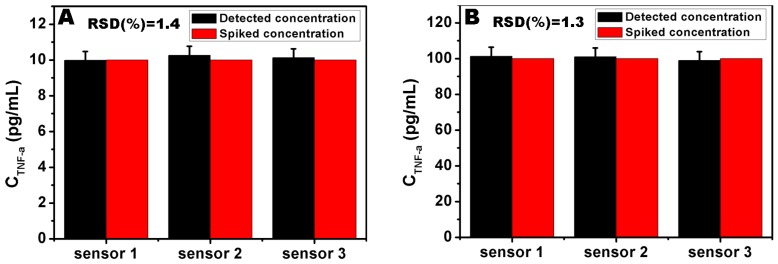
TNF-α was detected by the proposed sensor in the serum samples. The spiked concentration is (**A**) 10 pg/mL and (**B**) 100 pg/mL, respectively. (Relative standard deviation, RSD, *n* = 5).

In accordance with the above method, we further evaluated the detection of the serum samples. TNF-α at concentrations of 10 and 100 pg/mL was added to serum samples. After that, three serum samples were prepared for every concentration and were detected by our sensor. Moreover, every serum sample was detected three times. As shown in Figure 8, the average recoveries were 101.23% and 103.8%, respectively. The results indicated that this strategy was highly sensitive and that TNF-α was specifically recognized. This strategy thus has good prospects in clinical applications.

## 4. Conclusions

In sum, a novel, highly sensitive, reusable TNF-α sensor was successfully fabricated. The highly specific and affinity aptamer significantly improved detection precision and reduced the detection cost. The AuNPs-T3@DOX signal molecular greatly enhanced detection sensitivity. Compared with other sensors, our sensor is more sensitive, which changed linearly with TNF-α concentrations ranging from 1 to 10^4^ pg/mL, with the lowest detection limit of 0.7 pg/mL. Moreover, chain replacement reaction was cleverly utilized for the realization of the sensor reuse. The corresponding results indicated that the sensor could be reused up to six times. More importantly, the results of the serum sample detection revealed a strong potential for clinical applications. Therefore, our sensor may be an excellent method for the early detection of neonatal infections.

## Supplementary Materials

The following are available online at http://www.mdpi.com/1424-8220/17/5/992/s1. Figure S1: Sequences of the designed oligonucleotides; Table S1: Comparison of recently reported biosensors.
