# Supplementary material for: High Sensitivity Determination of TNF-α for Early Diagnosis of Neonatal Infections with a Novel and Reusable Electrochemical Sensor
Source: Sensors (Basel). 2017 May 10;17(5):992. doi: 10.3390/s17050992 (PMC5469345; doi:10.3390/s17050992)
Supplement: Supplementary file 1 [file sensors-17-00992-s001.pdf]

Supplementary Materials: High Sensitivity Determination of TNF-α for Early Diagnosis of Neonatal Infections with a Novel and Reusable Electrochemical Sensor

Liangliang Li, Miaomiao Li, Wenwen Wang, Qian Zhang, Dongyun Liu, Xianghong Li and Hong Jiang

Table S1. Sequences of the designed oligonucleotides.

Oligonucleotides	Sequences
Capture probe (T1)	5′-SH-TTGTCGCCGACTGCGCCATCC-3′
Aptamer (T2)	5′-TTTTTTTTTTTTTTTTGGTGGATGGCGCAGTCGGCGACAA-3′
Sgnal probe (T3)	5′- SH-GGATGGCGCAGT-3′

Table S2. Comparison of recently reported biosensors.

Method	Analyte	Line arrange	Detection limit	Reference
Fluorescence aptasensor	TNF-α	5–100 pg/mL		[1]
Electrochemical aptasensor	TNF-α	1–10^3 pg/mL	1 pg/mL	[2]
Electrochemical sensing	TNF-α	0–60 pg/mL	2.07 pg/mL	[3]
Electrochemical aptasensor	TNF-α	1–1 × 10^4 pg/mL	0.7 pg/mL	This work

1. Kongsuphol, P.; Ng, H.H.; Pursey, J.P.; Arya, S.K.; Wong, C.C.; Stulz, E.; Park, M.K. EIS-based biosensor for ultra-sensitive detection of TNF-*α* from non-diluted human serum. *Biosens. Bioelectron.* **2014**, *61*, 274–279.
2. Castro-López, V.; Elizalde, J.; Pacek, M.; Hijona, E.; Bujanda, L. A simple and portable device for the quantification of TNF-*α* in human plasma by means of on-chip magnetic bead-based proximity ligation assay. *Biosens. Bioelectron.* **2014**, *54*, 499–505.
3. Mazloum-Ardakani, M.; Hosseinzadeh, L.; Taleat, Z. Synthesis and electrocatalytic effect of Ag@Pt core-shell nanoparticles supported on reduced graphene oxide for sensitive and simple label-free electrochemical aptasensor. *Biosens. Bioelectron.* **2015**, *74*, 30–36.

© 2017 by the authors; licensee MDPI, Basel, Switzerland. This article is an open access article distributed under the terms and conditions of the Creative Commons by Attribution (CC BY) license (http://creativecommons.org/licenses/by/4.0/).
